# Genome Sequence of Pseudomonas sp. Strain So3.2b, Isolated from a Soil Sample from Robert Island (Antarctic Specially Protected Area 112), Antarctic

**DOI:** 10.1128/mra.01167-22

**Published:** 2023-02-21

**Authors:** Kattia Núñez-Montero, Dorian Rojas-Villalta, Ricardo Hernández-Moncada, Andrés Esquivel, Leticia Barrientos

**Affiliations:** a Centro de Investigación en Biotecnología, Instituto Tecnológico de Costa Rica, Cartago, Costa Rica; b Laboratorio de Biotecnología en Ambientes Extremos, Centro de Excelencia en Medicina Traslacional, Universidad de La Frontera, Temuco, Chile; Wellesley College

## Abstract

Strain So3.2b of the genus Pseudomonas was isolated from a soil sample from Robert Island (Antarctic Specially Protected Area 112), Antarctic. We report the complete genome sequence of this isolate, with a length of 6.17 Mbp and a GC content of 60.5%.

## ANNOUNCEMENT

Adverse and extreme conditions, such as those on the Antarctic continent, require specialized metabolites for microorganisms to adapt (1), which increases the interest in the discovery of natural products from extremophilic organisms (2). Here, we report the whole-genome sequence of Pseudomonas sp. strain So3.2b, a strain isolated from Antarctic soil.

Pseudomonas sp. strain So3.2b was obtained from a soil sample that had been collected in 2016 in Antarctic Specially Protected Area 112 (Robert Island [62.38S, 59.58W]), from a depth of 5 mm, with no surrounding vegetation or evidence of animal presence. Isolation was obtained by serial dilutions in Reasoner’s 2A (R2A) medium (Sigma-Aldrich, Darmstadt, Germany). Genomic DNA (gDNA) was obtained from the pure isolate grown in ISP-2 broth (4 g yeast extract, 10 g malt extract, 4 g glucose, 40 g mannitol) using the UltraClean microbial DNA extraction kit (MO BIO Laboratories, Carlsbad, CA, USA). Quality was determined with the QuantiFluor One double-stranded DNA (dsDNA) system (Promega), while integrity and purity were evaluated using 1% agarose gel electrophoresis plus *A*_260_/*A*_280_ and *A*_260_/*A*_230_ ratios.

The gDNA sample was split in two for hybrid sequencing and assembly using Illumina and Oxford Nanopore Technologies (ONT) (UK) systems. The Illumina gDNA library was prepared in 2 × 150-bp fragments using the Nextera XT DNA sample preparation kit (Illumina, San Diego, CA) and sequenced with an Illumina MiSeq system (Macrogen Inc., South Korea). The ONT gDNA library was prepared using the rapid sequencing gDNA SQK-RBK004 barcoding kit (ONT). The sequencing was carried out on a MinION system (Universidad de La Frontera, Chile) using a v9.4.1 flow cell with the software MinKNOW v4.0.20. ONT base calling was performed with Guppy v3.6.0 (3).

The quality of the raw Illumina reads was verified with FastQC v0.11.9 (4), showing a total of 7,620,395 sequences of 2 × 150-bp length for a sequencing depth of 185×. The reads were trimmed with fastp v0.20.1 (5). ONT read quality was determined with NanoPlot v1.40.2 (6), which resulted in a total of 136,219 reads with a read *N*_50_ value of 16,721 bp. Barcodes and low-quality reads (lengths of <500 bp and Q scores of <10) were trimmed using Porechop v0.2.4 and NanoFilt v2.8.0 (6), respectively. *De novo* assembly was carried out with both read libraries using Unicycler v0.4.8 (7), which allowed the confirmation of a circular sequence, followed by rotation of the genome to start at the *repA* gene, removal of overlapping sequences with the SPAdes optimizer v3.14.0, and genome polishing with Pilon v3.4. The assembly quality was determined using QUAST v5.0.2 (8), and its completeness and contamination were evaluated with CheckM v1.1.3 (9). Default parameters were used for data processing except where otherwise noted. Finally, the genome annotation was performed through the NCBI Prokaryotic Genome Annotation Pipeline (PGAP) v5.2 (10). We obtained a complete genome sequence of 6.17 Mb (Table 1).

**TABLE 1:** Characteristics and general sequencing results of Antarctic bacterial strain So3.2b

Parameter	Finding
Strain	Pseudomonas sp. strain So3.2b^*a*^
Sample source	Soil of Robert Island (Antarctic Specially Protected Area 112)
Genome size (bp)	6,167,549
GC content (%)	60.5
No. of contigs	1
No. of plasmids	0
Completeness (%)	94.29
Depth (×)	118
Total no. of coding sequences	5,513
Total no. of genes	5,402
No. of complete rRNAs (5S, 16S, 23S)	6, 5, 5
Total no. of tRNAs	66
Total no. of noncoding RNAs	4
Total no. of pseudogenes	111
Total no. of coding sequences without protein	111
BioProject accession no.	PRJNA605861
Genome accession no.	CP080494
Raw read information	
ONT library	
Total no. of reads	136,219
Total no. of bases	1,378,648,641
SRA accession number	SRX18029791
Illumina library	
Total no. of reads	7,620,395 (×2)
Total no. of bases	1,143,059,250 (×2)
SRA accession no.	SRX18029790

a Based on ANI comparison with available Pseudomonas whole-genome sequences in NCBI GenBank (accessed 20 October 2022).

The strain's identity, as determined by using BLASTn comparison (11) of the 16S rRNA sequence against the NCBI rRNA database, showed >99% similarity to multiple Pseudomonas species. The average nucleotide identity (ANI) values computed for 1,163 Pseudomonas complete genomes (available in the NCBI database [accessed 20 October 2022]) using FastANI v1.32 (12) showed the greatest similarity with Pseudomonas shahriarae (99.34%) and Pseudomonas fluorescens (99.08%), with no further results over the species threshold of 95%.

### Data availability.

The whole-genome shotgun project has been deposited in DDBJ/ENA/GenBank under the BioProject accession number PRJNA605861.
